# Supplementary material for: Why Are Some Male Alcohol Misuse Disorder Patients High Utilisers of Emergency Health Services? An Asian Qualitative Study
Source: Int J Environ Res Public Health. 2022 Aug 30;19(17):10795. doi: 10.3390/ijerph191710795 (PMC9518548; doi:10.3390/ijerph191710795)
Supplement: Supplementary file 1 [file ijerph-19-10795-s001.zip › ijerph-1813934-supplementary/File S1 - SNOMED-CT Codes.pdf]

Supplementary File S1: SNOMED-CT Codes

SNOMED ID	Term
101606018	Alcohol-induced psychotic disorder with delusions
10741871000119101	Alcohol dependence in pregnancy
10755041000119100	Alcohol dependence in childbirth
110628016	Alcohol dependence
110629012	Chronic alcoholism
115434017	Korsakoff's psychosis
115435016	Wernicke-Korsakoff syndrome
115436015	Amnestic syndrome of Wernicke's disease
121405015	Alcohol amnestic disorder
121406019	Alcohol-induced persisting amnestic disorder
1225776018	Alcoholic dementia
1233279015	Alcoholic hallucinosis
1234320010	Alcoholic peripheral neuropathy
12634015	Alcohol hallucinosis
12635019	Alcohol-induced psychotic disorder with hallucinations
12878014	Alcoholism
12879018	Alcoholism, NOS
133301000119102	Degenerative brain disorder caused by alcohol
135301000119103	Parasomnia caused by alcohol
135311000119100	Insomnia caused by alcohol
135321000119107	Hypersomnia caused by alcohol
135331000119105	Circadian rhythm sleep disorder caused by alcohol
136092014	Diarrhoea due to alcohol intake
14103017	Alcoholic polyneuropathy
14104011	Alcohol-induced polyneuropathy
141818010	Uncomplicated alcohol withdrawal
1490764012	Alcoholic cerebellar degeneration syndrome
15167005	Alcohol abuse
15243013	Alcohol withdrawal delirium
15244019	Delirium tremens
1557018	Dementia associated with alcoholism
1558011	Alcohol-induced persisting dementia
17389017	Acute alcoholic liver disease
17390014	Acute alcoholic hepatitis
18653004	Alcohol intoxication delirium
191471000	Korsakov's alcoholic psychosis with peripheral neuritis
191475009	Chronic alcoholic brain syndrome
191476005	Alcohol withdrawal hallucinosis
191477001	Pathological alcohol intoxication
191478006	Alcoholic paranoia

191480000	Alcohol withdrawal syndrome
191802004	Acute alcoholic intoxication in alcoholism
191804003	Continuous acute alcoholic intoxication in alcoholism
191805002	Episodic acute alcoholic intoxication in alcoholism
191806001	Acute alcoholic intoxication in remission, in alcoholism
191811004	Continuous chronic alcoholism
191812006	Episodic chronic alcoholism
191813001	Chronic alcoholism in remission
191882002	Nondependent alcohol abuse, continuous
191883007	Nondependent alcohol abuse, episodic
191884001	Nondependent alcohol abuse in remission
192811002	Alcoholic encephalopathy
19303008	Alcohol myopathy
2043009	Alcoholic gastritis
205788004	Fetal alcohol syndrome
205791004	Fetal or neonatal effect of maternal use of alcohol
21000000	Idiosyncratic intoxication
21007002	Wernicke's disease
212807002	Grain alcohol causing toxic effect
216633005	Accidental poisoning by alcoholic beverage
230353003	Morel laminar sclerosis
230800004	Alcoholic coma
231467000	Absinthe addiction
234341005	Alcohol-related macrocytosis
234366009	Alcohol-related sideroblastic anaemia
235875008	Alcoholic hepatitis
235880004	Alcoholic fibrosis and sclerosis of liver
235881000	Alcoholic hepatic failure
235942001	Alcohol-induced acute pancreatitis
235952002	Alcohol-induced chronic pancreatitis
237641009	Alcohol-induced hypoglycaemia
237738005	Alcohol-induced pseudo-Cushing's syndrome
2461892011	Accidental poisoning by alcoholic beverages (event)
2545415013	Alcoholic coma (disorder)
25702006	Alcohol intoxication
2573677012	Alcoholic cirrhosis (disorder)
25750014	Alcohol abuse
2576981019	Alcoholic cirrhosis
268645007	Nondependent alcohol abuse
268796000	Fetal or neonatal effect of placental or breast transfer of alcohol
278363000	Alcoholic macrocytosis
281004	Dementia associated with alcoholism
2817508011	Foetus and newborn affected by maternal use of alcohol

2820962019	Foetus OR newborn affected by alcohol transmitted via placenta AND/OR breast milk
2839024016	Korsakov alcoholic psychosis with peripheral neuritis
2839691016	Alcohol-induced pseudo-Cushing syndrome
2839693018	Wernicke disease
2840068011	Zieve syndrome
284591009	Persistent alcohol abuse
2868927014	Inflammation of pancreas due to alcohol (disorder)
2870612012	Alcohol-induced pancreatitis
2871112016	Inflammation of pancreas due to alcohol
288021000119107	Disorder due to alcohol abuse
288031000119105	Alcohol induced disorder co-occurrent and due to alcohol dependence
2912539019	Alcoholic liver cirrhosis
2920441014	Alcoholic cirrhosis of liver
29212009	Alcohol-induced organic mental disorder
294662017	Korsakov's alcoholic psychosis with peripheral neuritis
294668018	Chronic alcoholic brain syndrome
294669014	Alcohol withdrawal hallucinosis
294670010	Drunkenness - pathological
294671014	Pathological alcohol intoxication
294672019	Alcoholic paranoia
294674018	Alcohol withdrawal syndrome
295126010	Alcohol dependence with acute alcoholic intoxication
295127018	Acute alcoholic intoxication in alcoholism
295129015	Continuous acute alcoholic intoxication in alcoholism
295130013	Episodic acute alcoholic intoxication in alcoholism
295131012	Acute alcoholic intoxication in remission, in alcoholism
295139014	Continuous chronic alcoholism
295141010	Episodic chronic alcoholism
295142015	Chronic alcoholism in remission
2951649011	Alcohol induced mood disorder
2951733018	Alcohol induced encephalopathy
2951751017	Alcoholic pancreatitis
2951763010	Alcohol induced psychosis
2951773012	Alcohol induced sleep disorder
2951803016	Alcohol induced hallucinosis
2951813012	Alcohol induced anxiety disorder
2951840012	Alcohol induced psychosis with delusions
295252010	Nondependent alcohol abuse, continuous
295253017	Nondependent alcohol abuse, episodic
295254011	Nondependent alcohol abuse in remission
2965775012	Fetal or neonatal effect of placental or breast transfer of alcohol (disorder)
2965782011	Fetal or neonatal effect of maternal use of alcohol
2965793013	Fetal or neonatal effect of maternal use of alcohol (disorder)

2965854013	Fetal or neonatal effect of placental or breast transfer of alcohol
2965893016	Fetal or neonatal effect of maternal alcohol addiction (disorder)
2965922015	Foetal or neonatal effect of maternal alcohol addiction
2965936013	Fetal or neonatal effect of maternal alcohol addiction
2965947018	Fetal Alcohol Spectrum Disorder (disorder)
2965959019	Foetal Alcohol Spectrum Disorder
2966001015	Foetal or neonatal effect of placental or breast transfer of alcohol
2966136019	Fetal or neonatal effect of alcohol transmitted via placenta and/or breast milk
2966163018	Fetal or neonatal effect of alcohol transmitted via placenta and/or breast milk (disorder)
297011014	Alcoholic encephalopathy
2987871018	Neonatal effect of alcohol transmitted via breast milk
2988122010	Neonatal effect of alcohol transmitted via breast milk (disorder)
2990705017	Alcohol dependence in pregnancy (disorder)
2990708015	Alcohol dependence in pregnancy
2995248010	Alcohol dependence in childbirth (disorder)
2995391019	Alcohol dependence in childbirth
300992002	Alcohol-induced cerebellar ataxia
3010279011	Foetal alcohol syndrome
3011881012	FAS - Foetal alcohol syndrome
3012421016	Foetal or neonatal effect of alcohol transmitted via placenta and/or breast milk
3027285017	Alcoholic fatty liver disease
3037101014	Pseudo-Cushing syndrome due to alcohol
307757001	Chronic alcoholic hepatitis
3078939011	Chronic pancreatitis due to acute alcohol intoxication
308692003	Alcohol-induced flushing
309783001	Oesophageal varices in alcoholic cirrhosis of the liver
31451012	Alcohol intoxication delirium
315571015	Fetal alcohol syndrome
315572010	FAS - Fetal alcohol syndrome
315575012	Fetus and newborn affected by maternal use of alcohol
324466015	Grain alcohol causing toxic effect
32551012	Alcohol myopathy
32552017	Alcoholic myopathic syndrome
32553010	Alcoholic myositis
328738016	Accidental poisoning by alcoholic beverages
3288358017	Chronic alcoholic liver disease (disorder)
3288362011	Chronic alcoholic liver disease
3289052015	Acute on chronic alcoholic liver disease (disorder)
3289055018	Acute on chronic alcoholic liver disease
3289369011	Thrombocytopaenia co-occurrent and due to alcoholism
3289384019	Thrombocytopenia co-occurrent and due to alcoholism (disorder)
3289961017	Degenerative brain disorder caused by alcohol (disorder)
3289963019	Circadian rhythm sleep disorder caused by alcohol

3289991010	Insomnia caused by alcohol
3289992015	Hypersomnia due to alcohol
3290021010	Degenerative brain disorder caused by alcohol
3290034011	Hypersomnia caused by alcohol
3290047018	Circadian rhythm sleep disorder caused by alcohol (disorder)
3290063014	Hypersomnia caused by alcohol (disorder)
3290078019	Parasomnia caused by alcohol (disorder)
3290091018	Degenerative brain disorder due to alcohol
3290095010	Insomnia caused by alcohol (disorder)
3290111016	Parasomnia due to alcohol
3290112011	Insomnia due to alcohol
3290119019	Parasomnia caused by alcohol
3290136010	Disorder due to alcohol abuse (disorder)
3290157019	Disorder due to alcohol abuse
3290326011	Mild alcohol dependence (disorder)
3290336015	Mild alcohol dependence
3290967013	Inflammation of pancreas caused by alcohol (disorder)
3291507014	Inflammation of pancreas caused by alcohol
3293636013	Diarrhea caused by alcohol intake (disorder)
3293983012	Megaloblastic anemia caused by alcoholism (disorder)
3294127015	Megaloblastic anaemia caused by alcoholism
3295289018	Diarrhoea caused by alcohol intake
3297729017	Severe alcohol dependence (disorder)
3297813018	Severe alcohol dependence
3300566013	Moderate alcohol dependence (disorder)
3300567016	Moderate alcohol dependence
3300760016	Accidental poisoning caused by alcoholic beverage (disorder)
3300761017	Accidental poisoning caused by alcoholic beverage
3300762012	Accidental poisoning by alcoholic beverage
3316454015	Alcohol induced disorder co-occurrent and due to alcohol dependence (disorder)
3316455019	Alcohol induced disorder co-occurrent and due to alcohol dependence
3316456018	Alcohol dependence with alcohol induced disorder
3325821017	Fibrosis of liver caused by alcohol (disorder)
3325822012	Fibrosis of liver caused by alcohol
3325823019	Alcoholic fibrosis of liver
3334613012	Gastric ulcer caused by alcohol (disorder)
3334614018	Gastric ulcer caused by alcohol
3334615017	Alcohol induced gastric ulcer
3426713010	Duodenitis caused by ingestible alcohol (disorder)
3426714016	Duodenitis caused by ingestible alcohol
3426715015	Alcoholic duodenitis
3427867017	Psychotic disorder caused by alcohol with schizophreniform symptoms (disorder)
3427868010	Psychotic disorder caused by alcohol with schizophreniform symptoms

3427869019	Alcohol-induced psychotic disorder with schizophreniform symptoms
3427875011	Perceptual disturbances and seizures co-occurrent and due to alcohol withdrawal (disorder)
3427876012	Perceptual disturbances and seizures co-occurrent and due to alcohol withdrawal
3427877015	Alcohol withdrawal with perceptual disturbances and seizures
3429894014	Mood disorder with mixed manic and depressive symptoms caused by alcohol (disorder)
3429895010	Mood disorder with mixed manic and depressive symptoms caused by alcohol
3429896011	Alcohol-induced mood disorder with mixed manic and depressive symptoms
3429897019	Mood disorder with manic symptoms caused by alcohol (disorder)
3429898012	Mood disorder with manic symptoms caused by alcohol
3429899016	Alcohol-induced mood disorder with manic symptoms
3429900014	Mood disorder with depressive symptoms caused by alcohol (disorder)
3429901013	Mood disorder with depressive symptoms caused by alcohol
3429902018	Alcohol-induced mood disorder with depressive symptoms
3441483014	Neurological disorder caused by ingestible alcohol (disorder)
3441484015	Neurological disorder caused by ingestible alcohol
3441485019	Alcohol-related neurological disorder
345186013	Morel laminar sclerosis
345805013	Alcoholic coma
346934011	Absinthe addiction
34938008	Alcohol-induced anxiety disorder
351066013	Alcohol-related macrocytosis
351121019	Alcohol-related sideroblastic anaemia
35303013	Idiosyncratic intoxication
35304019	Pathological alcohol intoxication
35305018	Extreme sensitivity to alcohol syndrome
35313017	Wernicke's disease
35314011	Wernicke's encephalopathy
353587018	Alcoholic hepatitis
353588011	AH - Alcoholic hepatitis
353594015	Alcoholic fibrosis and sclerosis of liver
353595019	Alcoholic hepatic failure
353687012	Alcohol-induced acute pancreatitis
353698013	Alcohol-induced chronic pancreatitis
356162012	Alcohol-induced hypoglycaemia
356292013	Alcohol-induced pseudo-Cushing's syndrome
361268000	Alcohol-induced epilepsy
361272001	Cerebellar ataxia due to alcoholism
361273006	Alcoholic cerebellar degeneration
36558000	Fetal or neonatal effect of alcohol transmitted via placenta and/or breast milk
3658935010	Steatohepatitis caused by ingestible alcohol
3658936011	Steatohepatitis caused by ingestible alcohol (disorder)
3658938012	Alcoholic steatohepatitis
401797010	Nondependent alcohol abuse

402169011	Fetus or neonate affected by placental or breast transfer of alcohol
41083005	Alcohol-induced sleep disorder
41309000	Alcoholic liver damage
415240012	Alcoholic macrocytosis
420054005	Alcoholic cirrhosis
423307016	Persistent alcohol abuse
423308014	Chronic alcohol abuse
42344001	Alcohol-induced psychosis
43081018	Alcohol intoxication
43082013	Acute alcoholism
43083015	Drunkenness
44047000	Zieve's syndrome
442200018	Alcohol-induced cerebellar ataxia
445507008	Alcohol-induced pancreatitis
451124014	Chronic alcoholic hepatitis
452014017	Alcohol-induced flushing
452015016	Alcohol-induced flushes
4525015	Alcoholic gastritis
453265010	Oesophageal varices in alcoholic cirrhosis of the liver
477198011	Ethanol abuse
477199015	AA - Alcohol abuse
478024019	Alcohol-induced epilepsy
478046010	Cerebellar ataxia due to alcoholism
478048011	Alcoholic cerebellar degeneration
483024013	Acute alcohol intoxication
483025014	Drunk
48886010	Alcohol-induced organic mental disorder
48887018	Alcohol-induced organic mental disorder, NOS
48888011	Alcohol-related disorder, NOS
48889015	Alcohol-related disorder
492959019	ALD - Alcoholic liver disease
500478011	Alcohol dependence syndrome
501294012	Wernicke-Korsakov syndrome
501295013	Korsakoff psychosis
501296014	Korsakov alcoholic psychosis
501297017	Korsakov syndrome - alcoholic
501298010	Korsakov psychosis
501299019	Amnesic syndrome due to alcohol
501300010	Alcoholic amnestic syndrome
502016018	Dipsomania
502017010	Alcohol problem drinking
50325005	Alcoholic fatty liver
507053019	DTs - delirium tremens

507760015	Megaloblastic anaemia due to alcoholism
53936005	Alcohol-induced mood disorder
575174011	Korsakov's alcoholic psychosis with peripheral neuritis (disorder)
575178014	Chronic alcoholic brain syndrome (disorder)
575179018	Alcohol withdrawal hallucinosis (disorder)
575180015	Pathological alcohol intoxication (disorder)
575181016	Alcoholic paranoia (disorder)
575183018	Alcohol withdrawal syndrome (disorder)
575539019	Acute alcoholic intoxication in alcoholism (disorder)
575541018	Continuous acute alcoholic intoxication in alcoholism (disorder)
575542013	Episodic acute alcoholic intoxication in alcoholism (disorder)
575543015	Acute alcoholic intoxication in remission, in alcoholism (disorder)
575549016	Continuous chronic alcoholism (disorder)
575551017	Episodic chronic alcoholism (disorder)
575552012	Chronic alcoholism in remission (disorder)
575628013	Nondependent alcohol abuse, continuous (disorder)
575629017	Nondependent alcohol abuse, episodic (disorder)
575630010	Nondependent alcohol abuse in remission (disorder)
576633016	Alcoholic encephalopathy (disorder)
58300018	Alcohol-induced anxiety disorder
591204015	Fetal alcohol syndrome (disorder)
591207010	Fetus and newborn affected by maternal use of alcohol (disorder)
598679014	Grain alcohol causing toxic effect (disorder)
602910010	Accidental poisoning by alcoholic beverages (disorder)
609437000	Foetal Alcohol Spectrum Disorder
609438005	Fetal or neonatal effect of maternal alcohol addiction
61010014	Fetus OR newborn affected by alcohol transmitted via placenta AND/OR breast milk
61011013	Fetus or newborn affected by alcohol transmitted via placenta or breast milk
61012018	Fetal alcohol syndrome
61013011	Foetal alcohol syndrome
61144001	Alcohol-induced psychotic disorder with delusions
618149013	Morel laminar sclerosis (disorder)
618656015	Alcoholic coma (finding)
619402011	Absinthe addiction (disorder)
622647017	Alcohol-related macrocytosis (disorder)
622674016	Alcohol-related sideroblastic anemia (disorder)
624392019	Alcoholic hepatitis (disorder)
624397013	Alcoholic fibrosis and sclerosis of liver (disorder)
624399011	Alcoholic hepatic failure (disorder)
624469018	Alcohol-induced acute pancreatitis (disorder)
624480018	Alcohol-induced chronic pancreatitis (disorder)
626399013	Alcohol-induced hypoglycemia (disorder)
626510012	Alcohol-induced pseudo-Cushing's syndrome (disorder)

661410013	Nondependent alcohol abuse (disorder)
661578014	Fetus or neonate affected by placental or breast transfer of alcohol (disorder)
66590003	Alcohol dependence
671509016	Alcoholic macrocytosis (disorder)
678501012	Persistent alcohol abuse (disorder)
68536019	Alcohol-induced sleep disorder
68906017	Alcoholic liver damage
68907014	Alcoholic liver damage, NOS
68908016	Alcoholic liver disease, NOS
68909012	Alcoholic liver disease
69482004	Korsakoff's psychosis
696735013	Alcohol-induced cerebellar ataxia (disorder)
698321001	Neonatal effect of alcohol transmitted via breast milk
704320014	Chronic alcoholic hepatitis (disorder)
705197018	Alcohol-induced flushing (disorder)
7052005	Alcohol hallucinosis
706333018	Esophageal varices in alcoholic cirrhosis of the liver (disorder)
70655012	Alcohol-induced psychosis
70656013	Alcohol-induced psychosis, NOS
70657016	Alcoholic psychosis, NOS
70658014	Alcoholic psychosis
713181003	Chronic alcoholic liver disease
713370005	Acute on chronic alcoholic liver disease
713583005	Mild alcohol dependence
713862009	Severe alcohol dependence
714829008	Moderate alcohol dependence
7200002	Alcoholism
721710005	Fibrosis of liver caused by alcohol
723103002	Gastric ulcer caused by alcohol
723882007	Duodenitis caused by ingestible alcohol
723926008	Perceptual disturbances and seizures co-occurrent and due to alcohol withdrawal
723927004	Psychotic disorder caused by alcohol with schizophreniform symptoms
723928009	Mood disorder with depressive symptoms caused by alcohol
723929001	Mood disorder with manic symptoms caused by alcohol
723930006	Mood disorder with mixed manic and depressive symptoms caused by alcohol
724574008	Neurological disorder caused by ingestible alcohol
73097000	Alcohol amnestic disorder
73449014	Zieve's syndrome
741908010	Alcohol abuse (disorder)
744130013	Alcohol-induced epilepsy (disorder)
744179017	Cerebellar ataxia due to alcoholism (disorder)
744191014	Alcoholic cerebellar degeneration (disorder)
746176010	Alcohol intoxication delirium (disorder)

747279016	Alcohol myopathy (disorder)
749579012	Alcoholic gastritis (disorder)
750245012	Idiosyncratic intoxication (disorder)
750254010	Wernicke's disease (disorder)
756051015	Alcohol intoxication (disorder)
758743015	Dementia associated with alcoholism (disorder)
760082013	Alcohol-induced organic mental disorder (disorder)
765482002	Alcoholic steatohepatitis
766520019	Alcohol-induced anxiety disorder (disorder)
768323014	Fetus OR newborn affected by alcohol transmitted via placenta AND/OR breast milk (disorder)
777831012	Alcohol-induced sleep disorder (disorder)
778083018	Alcoholic liver damage (disorder)
779233014	Alcohol-induced psychosis (disorder)
781130011	Zieve's syndrome (disorder)
788101014	Alcoholic fatty liver (disorder)
7916009	Alcoholic polyneuropathy
792111010	Alcohol-induced mood disorder (disorder)
800174015	Alcohol-induced psychotic disorder with delusions (disorder)
806219017	Alcohol dependence (disorder)
809432012	Korsakoff's psychosis (disorder)
810586018	Alcohol hallucinosis (disorder)
812229019	Alcoholism (disorder)
813447015	Alcohol amnestic disorder (disorder)
820180017	Alcoholic polyneuropathy (disorder)
82047000	Diarrhoea due to alcohol intake
823387011	Diarrhea due to alcohol intake (disorder)
827641011	Uncomplicated alcohol withdrawal (disorder)
828596013	Alcohol withdrawal delirium (disorder)
830362014	Megaloblastic anemia due to alcoholism (disorder)
83834016	Alcoholic fatty liver
844545010	Acute alcoholic liver disease (disorder)
85561006	Uncomplicated alcohol withdrawal
8635005	Alcohol withdrawal delirium
87810006	Megaloblastic anaemia due to alcoholism
89659017	Alcohol-induced mood disorder
97571000119109	Thrombocytopaenia co-occurrent and due to alcoholism
9953008	Acute alcoholic liver disease
