# Supplementary material for: Why Are Some Male Alcohol Misuse Disorder Patients High Utilisers of Emergency Health Services? An Asian Qualitative Study
Source: Int J Environ Res Public Health. 2022 Aug 30;19(17):10795. doi: 10.3390/ijerph191710795 (PMC9518548; doi:10.3390/ijerph191710795)
Supplement: Supplementary file 1 [file ijerph-19-10795-s001.zip › ijerph-1813934-supplementary/File S2 - Interview Questionnaire.pdf]

Supplementary File S2: Interview questionnaire

SECTION 1: PERSONAL INFORMATION AND BACKGROUND

Section 1 to be* ***filled in by participants after the interview* *should this information not be retrievable from hospital database.*

1. Gender
   - I. Male
   - II. Female
   - III. Others (Please state: ________)
2. Age
   - I. Open-ended (state number of years old)
   - II. When did you first started drinking?
3. Race
   - I. Chinese
   - II. Malay
   - III. Indian
   - IV. Caucasian
   - V. Others (Please state: ____________
4. Religion
   - I. Buddhist
   - II. Christian
   - III. Islam
   - IV. Hinduism
   - V. Catholic
   - VI. Taoism
   - VII. Others (Please state: ________)

5. Relationship status

   I. Single

   II. Married

   III. Separated

   IV. Divorced

   V. Widowed

6. Housing

   I. Homeless

   II. One/two room HDB

   III. Three/four room HDB

   IV. Five room HDB/Executive Condominium

   V. Private housing

   VI. Sheltered or Nursing Home

7. Education

   I. None

   II. Primary

   III. Secondary

   IV. Pre-tertiary (ITE, Polytechnic, JC)

   V. Tertiary

8. What is your monthly income level based on current employment?

   I. No salary

   II. $<1000

   III. $1000-2000

   IV. $ 2000-3000

   V. $3000 - 5000

   VI. > $5000

9. Smoker

   I. Yes/No

II. Age started

III. How many sticks/day

IV. If stopped, for how long

10. Prison

    I. If yes, how long and for what reason?

11. Family structure and relationships

    I. What is your relationship with your family? (5-Point Likert Scale; 1 = Very distant, 5 = Very close)

12. Friendship

    I. What is your relationship with your friends? (5-Point Likert Scale; 1 = Very distant, 5 = Very close)

SECTION 2a: DETERMINATION OF PERSONAL ALCOHOLISM PROFILE

Question	Response set (Choose only one)	Rationale
1. During the last 12 months, how often did you usually have any kind of drink containing alcohol? **By a drink we mean (e.g. a glass of beer or cooler, a glass of wine, or a drink containing 1 shot of liquor)**.	Every day<br>5 to 6 times a week<br>3 to 4 times a week<br>twice a week<br>once a week<br>2 to 3 times a month<br>once a month<br>3 to 11 times in the past year<br>1 or 2 times in the past year<br><br>(IF RESPONDENT GIVES ANY OF THE ABOVE	This question is from the National Institute on Alcohol Abuse and Alcoholism (NIAAA) 6 question set of recommended alcohol questions

	RESPONSES, GO TO QUESTION 2)<br><br>I did not drink any alcohol in the past year, but I did drink in the past<br>(GO TO QUESTION 1A)<br><br>I never drank any alcohol in my life<br>(GO TO QUESTION 1B)	
**1A** - During your lifetime, what is the maximum number of drinks containing alcohol that you drank within a 24-hour period? (asked here only of those who did not drink any alcohol during the past 12 months)	36 drinks or more<br>24 to 35 drinks<br>18 to 23 drinks<br>12 to 17 drinks<br>8 to 11 drinks<br>5 to 7 drinks<br>4 drinks<br>3 drinks<br>2 drinks<br>1 drink<br>(DONE WITH ALCOHOL QUESTIONS)	This question is from NIAAA 6 question set of recommended alcohol questions
**1B** - So you have never had a drink containing alcohol in your entire life. (asked only of those who say they	Yes, I never drank.<br>(DONE WITH ALCOHOL QUESTIONS)	This question is from NIAAA 6 question set of recommended alcohol questions

never drank alcohol in their lives)	No, I did drink<br>(GO BACK TO QUESTION 1 AND REPEAT)	
2. During the last 12 months, how many alcoholic drinks did you have on a typical day when you drank alcohol?<br><br>2a. [Where relevant] Did you drink more in the last 12 months during COVID-19? Why did you drink more?	25 or more drinks<br>19 to 24 drinks<br>16 to 18 drinks<br>12 to 15 drinks<br>9 to 11 drinks<br>7 to 8 drinks<br>5 to 6 drinks<br>3 to 4 drinks<br>2 drinks<br>1 drink	This question is from NIAAA 6 question set of recommended alcohol questions
3. During the last 12 months, what is the largest number of drinks containing alcohol that you drank within a 24-hour period?	36 drinks or more<br>24 to 35 drinks<br>18 to 23 drinks<br>12 to 17 drinks<br>8 to 11 drinks<br>5 to 7 drinks<br>4 drinks<br>3 drinks<br>2 drinks<br>1 drink	This question is from NIAAA 6 question set of recommended alcohol questions

4. During the last 12 months, how often did you drink this largest number of drinks? Choose only one.	Every day<br>5 to 6 times a week<br>3 to 4 times a week<br>twice a week<br>once a week<br>2 to 3 times a month<br>once a month<br>3 to 11 times in the past year<br>1 or 2 times in the past year	This question is from NIAAA 6 question set of recommended alcohol questions
5. During the last 12 months, how often did you have 5 or more (males) or 4 or more (females) drinks containing any kind of alcohol in within a two-hour period? [That would be the equivalent of at least 5 (4) cans or bottles of beer, 5 (4) glasses of wine, 5 (4) drinks each containing one shot of liquor or spirits - to be provided by interviewer if asked.]	Every day<br>5 to 6 days a week<br>3 to 4 days a week<br>two days a week<br>one day a week<br>2 to 3 days a month<br>one day a month<br>3 to 11 days in the past year<br>1 or 2 days in the past year	This question is from NIAAA 6 question set of recommended alcohol questions

6. During your lifetime, what is the largest number of drinks containing alcohol that you drank within a 24-h/our period?	36 drinks or more<br>24 to 35 drinks<br>18 to 23 drinks<br>12 to 17 drinks<br>8 to 11 drinks<br>5 to 7 drinks<br>4 drinks<br>3 drinks<br>2 drinks<br>1 drink	This question is from NIAAA 6 question set of recommended alcohol questions
7. What do you often drink (type of alcohol consumed) and why?	Open-ended	
8. What triggers you to drink?<br>8a. In the past, what triggered you to drink? How did you increase from 1 to 2 drinks in the past to this many today? What happened?	Open-ended	
9. How do you feel when you drink?	Open-ended	To determine any pleasure/discomfort associated with drinking behaviours.

9a. How do you feel *before* drinking?<br><br>9b. How did you feel *during* drinking?<br><br>9c. How did you feel *after* drinking?		
10. What are some obstacles you face with your drinking behavior?	Unsustainable cost<br><br>Negative perception<br><br>Physical ailments (including withdrawal)<br><br>Emotional distress (including withdrawal)	To identify the presence of protective factors.

SECTION 2b: DETERMINATION OF SOCIAL ALCOHOLISM PROFILE

Question	Response set	Rationale
1. Do people in your social circle (i.e., family, friends) drink? Do you drink with them?	Yes/No<br><br>Yes/No. Specify who.	To identify if the consumption of alcohol is a socially learnt behaviour.

2. How do you think they feel about your drinking behaviours?	Open-ended	To identify if the respondent's social environment enables his/her addiction.

SECTION 3: USE OF EMERGENCY MEDICAL SERVICES

Question	Response set	Rationale
1. How often do you call the emergency services in a month?	Open-ended	*Question to be asked only if information cannot be released by SCDF/hospital
2. How often do you get warded?	Open-ended	To identify the services used by respondents when they call emergency services.<br><br>*Question to be asked only if information cannot be released by SCDF/hospital
3. Why do you call emergency services?	Open-ended<br><br>Possible answers include physical discomfort, emotional distress, loneliness, boredom, excitement, ambivalence. If	To identify factors behind participant's use of emergency services

	necessary, can use these as options for respondent.	
4. After getting a response from the emergency services, how do you feel? Why do you feel this way?	5-Point Likert scale (1 = Very Bad, 3 = Ambivalent, 5 = Very good)<br><br>Open-ended	To identify if there are any emotional factors that might inhibit further use of emergency services.
5. What cases do you think the A&E deals with?	Open-ended	To tease out a general perception of the A&E (i.e., what kinds of cases are admitted to the A&E, what the purpose of the A&E is).

SECTION 4a: MOTIVATION BEHIND CORRECTIVE BEHAVIOURS/ATTEMPTS & ATTITUDES TOWARDS INTERVENTIONS

Questions	Response set	Rationale
1. Have you ever felt you needed to cut down on your drinking? If yes, what problems have you faced when trying?	Open-ended	To identify any prior attempts to stop the respondent's drinking addiction.

2. What is your goal when it comes to your drinking habits?	Stopping completely<br>Reducing intake<br>Addressing root cause of the problem<br>N/A	To discern level of self-motivation and self-awareness
3. Have you received help for your drinking? If yes, where did you received help from? If no, why not?	Prompts: WE CARE, NAMS, The Cabin Singapore	
4. What are your feelings towards seeking treatment?	Prompts: proud, disappointed, ambivalent	To find out if there are any internal motivating factors that keep respondent in treatment.
5. How do you feel **during** treatment?	5-Point Likert scale (1 = Very Bad, 3 = Ambivalent, 5 = Very good)	To find out if there are any internal motivating factors that keep respondent in treatment.
6. How are people in your life reacting to you seeking treatment?	Open-ended	To find out if respondent has any external motivating factors that push him/her to seek

		treatment or keep him/her in treatment.
7. Do you feel like the interventions helped you with curbing your drinking behaviours?	Yes/No	To find out respondent's experience with the help received for his/her alcoholism.
8. How do you find the professional capabilities of your healthcare providers?	5-Point Likert scale (1 = Very bad, 5 = Very good)	To gauge respondent's interaction with healthcare professionals and whether it is conducive/effective for recovery.
